# Influences of different fiber-reinforced biobases on the fracture strength and failure mode of lithium disilicate overlay restorations (a comparative *in vitro* study)

**DOI:** 10.4317/jced.62746

**Published:** 2025-08-01

**Authors:** Maareb Abdulraheem Nabat, Alaa Jawad Kadhim

**Affiliations:** 1 B.D.S. Department of Restorative and Aesthetic Dentistry, College of Dentistry, University of Baghdad, Baghdad, Iraq; 2 B.D.S., Ms.c. Department of Restorative and Aesthetic Dentistry, College of Dentistry, University of Baghdad, Baghdad, Iraq

## Abstract

**Background:**

Ceramic restorations are commonly employed in restorative dentistry. However, their inherent brittleness poses a challenge, particularly in extensive restorations. Limited data exist regarding the fiber reinforcement’s role in the efficiency of lithium disilicate overlay restorations. This research sought to evaluate the fracture strength and failure mode of lithium disilicate overlay restorations using various biobase techniques.

**Material and Methods:**

Fifty sound maxillary first premolars of equivalent dimensions received a full-bevel overlay preparation design with a 3-mm occlusal reduction. Samples were allocated randomly to five experimental groups (n = 10): Group A: (delayed dentin sealing); Group B: (immediate dentin sealing with Optibond FL); Group C: immediate dentin sealing was coated with a 1-mm flowable composite layer (Clearfil AP-X Flow); Group D: following immediate dentin sealing, a 1-mm short fiber reinforced composite layer (EverX Flow) was applied; and Group E: immediate dentin sealing followed by a 1-mm flowable composite layer reinforced with polyethylene Ribbond fibers. Following tooth preparation, digital impressions were made via a Medit i700 intraoral scanner, and overlays were digitally designed via Sirona inLab CAD software and milled via a 4-axis milling machine. The overlays were luted with a preheated composite. The fracture load was assessed (in Newtons) utilizing a universal testing unit. A one-way analysis of variance was employed to perform statistical analysis.

**Results:**

The one-way ANOVA test indicated no significant difference between the groups (*P*> 0.05). Nevertheless, the fiber-reinforced biobases in Groups D and E exhibited less catastrophic failure modes than those in Groups A, B, and C.

**Conclusions:**

All of the tested overlay restorations displayed sufficient strength to endure the normal masticatory force. The incorporation of fiber-reinforced biobases positively influenced the failure mode.

** Key words:**Biobase, Ever X flow, Full bevel, Overlay, Ribbond fiber.

## Introduction

The restoration of posterior teeth with substantial coronal defects remains a common issue in dentistry, with several treatment options available to address this issue [1]. While direct restorations are often the first choice because of their ability to preserve the tooth structure, indirect restorations offer the advantages of enhanced tooth contour, anatomic form, improved occlusal contacts, and superior mechanical characteristics [2].

Among indirect restorations, partial coverage overlay restorations are regarded as a conservative alternative to full crown restorations, offering a balance between functional restoration and structural preservation [1].

Glass ceramic materials, especially lithium disilicate, are commonly preferred for indirect restorations owing to their superior mechanical properties and aesthetic appeal [3,4]. They bonded adhesively to dentin; the immediate dentin sealing (IDS) was intended to improve their adhesion. IDS can be performed using the following two approaches:

• The dentin bonding agent is applied immediately following cavity preparation [5].

• A thin film of low viscosity flowable composite resin is positioned atop the bonded dentin [6].

A biobase is a highly bonded basis that reduces stress; and serves as the bonding interface for indirect restorations. According to biomimetic dentistry school protocol, a biobase consists of deep margin elevation, IDS, a resin coat (RC), and a dentin-replacing composite [7].

Fracture toughness is a key material property that represents a brittle material’s ability to withstand the catastrophic spread of fissures when exposed to an external load. As such, it is an essential indicator of the material’s capacity to tolerate damage [8].

Despite their strength, composite and lithium disilicate materials exhibit inherent brittleness and insufficient toughness [9]. This limitation becomes particularly critical in extensive restorations in which brittle materials replace large amounts of dentin, which has higher fracture toughness than these restorative materials [10].

To overcome this challenge, materials with increased fracture toughness have been proposed as ideal substitutes for the lost dentin‒enamel junction (DEJ) and dentin core [11]. Fiber reinforcement has emerged as a promising solution, with the biomimetic protocol advocating the use of leno wave ultrahigh-molecular-weight polyethylene (UHMWPE) Ribbond fibers to reinforce the compromised tooth structure and dissipate occlusal stresses [12]. Additionally, short fiber reinforced composite (SFRC) material, characterized by the incorporation of multidirectional fibers, has demonstrated fracture toughness comparable to natural dentine, making it a viable substitute for dentin in structurally compromised teeth [11].

However, existing research has primarily focused on using fiber reinforcement beneath direct composite restorations, leading to noTable improvements in the fracture strength and failure modes [13,14]. Limited data exist regarding its impact on indirect lithium disilicate overlay restorations. Hence, this research focused on bridging this gap by examining the fracture strength and fracture modes of overlay restorations utilizing different biobase techniques.

## Material and Methods

- Tooth selection and grouping

The Committee of Research Ethics of Baghdad University, College of Dentistry, Iraq, approved this study in January 2024 (Approval No. 895524). A total of fifty maxillary first premolars with comparable dimensions extracted for orthodontic purposes were obtained from individuals between 18 and 22 years of age. The tooth samples were distributed at random to five groups (*n*=10) using a computer-generated simple randomization method created in Microsoft Excel based on the biobase technique utilized: Group A: delayed dentin sealing (DDS); Group B: IDS; and Group C: IDS plus RC; Group D: IDS with a SFRC biobase; and Group E: IDS with a Ribbond fiber-reinforced biobase.

- Specimen preparation and biobase application

**Figure 1:**
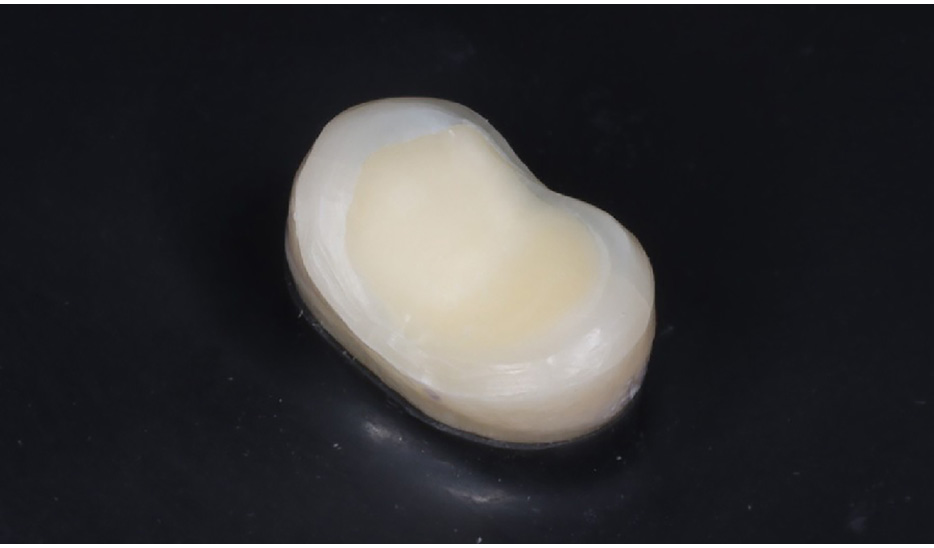
The prepared tooth surface.

**Figure 2:**
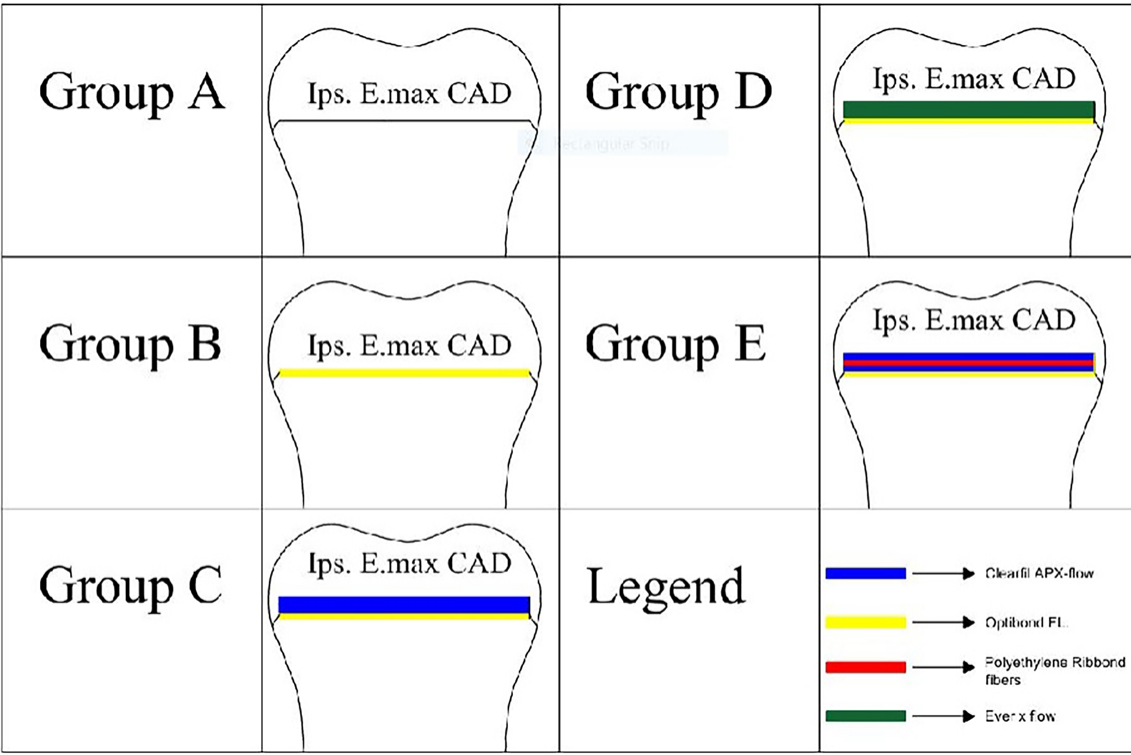
The experimental groups.

A standardized full-bevel preparation design was utilized for all the samples to accommodate indirect overlay restorations, with an occlusal reduction of 3 mm, as shown in Fig. 1. Following cavity preparation, dentin sealing was performed using Optibond FL (Kerr, Italy) immediately in all groups except Group A (DDS), where dentin sealing was intentionally delayed. IDS was followed for Group C by applying a 1-mm microfilled flowable composite layer (Clearfil AP-X Flow, Kuraray, Tokyo, Japan) over the sealed dentin, in Group D by incorporating a 1-mm SFRC layer (Ever X flow/Dentin shade, GC Europe, Belgium) over the sealed dentin, and in Group E by initially applying approximately 0.5 mm of flowable composite layer (Clearfil AP-X Flow) atop the sealed dentin and leaving it uncured. Ribbond fiber of a suiTable buccolingual length presoaked in Ribbond Wetting Resin (Ribbond Ultra, Ribbond Inc., Seattle WA, USA) was subsequently applied atop the dentin surface along the buccolingual direction and light-cured for 20 secs. A second flowable composite film was added over the fiber-reinforced composite layer and light cured, ensuring complete encapsulation of the exposed fibers and a final biobase thickness of 1 mm, as shown in Fig. 2.

- Overlay fabrication and cementation

A Medit i700 intraoral scanner (Korea) was utilized for the prepared teeth scanning. Then, temporary restorative material (Revotek LC, GC, Tokyo, Japan) was applied. The overlay restorations were digitally designed via Sirona inLab CAD software (version 20.0) and subsequently milled from (IPS e.max CAD, Ivoclar Vivadent) lithium disilicate blocks via an inLab MC XL 4-axis milling machine. The milled overlays were exposed to crystallization and glaze firing in a P500 furnace at 840 °C.

The restorations’ internal surfaces underwent 20 seconds of etching using hydrofluoric acid (etching gel > 5%, Ivoclar Vivadent), followed by a 15-second water rinse. Then, they were immersed in a 90% alcohol ultrasonic bath for 5 minutes to remove etching remnants. Afterward, a silane (Bisco, USA) was applied for 20 seconds, and the mixture was subjected to drying at 100 °C (212 °F) for 2 minutes using a hair dryer.

After removing the provisional restoration, the prepared tooth surfaces were cleaned and reactivated through an AquaCare airborne particle abrasion device (UK). The surfaces were treated with (50-µm) aluminum oxide airborne particles for 5 seconds under 15- and 2-mm bar pressures.

Next, phosphoric acid 37% (Kerr, Italy) was applied to all the samples for 30 s, after which they were rinsed thoroughly and dried for 20 and 3 s. Only the samples in Group A (DDS) received an additional primer application for 15 seconds, then dried for 5 seconds. An adhesive resin was gently brushed for 15 s without polymerization. The restorations were then cemented using a preheated microhybrid composite (Clearfil AP-X, Kuraray, Tokyo, Japan), which had been heated in an Ena heat composite heater (Micerium, Italy) at 68°C for 15 minutes. Cementation was performed using a custom-made specimen holding device, ensuring a standardized 5-kg vertical load for precise bonding [15].

- Fracture strength test

**Figure 3:**
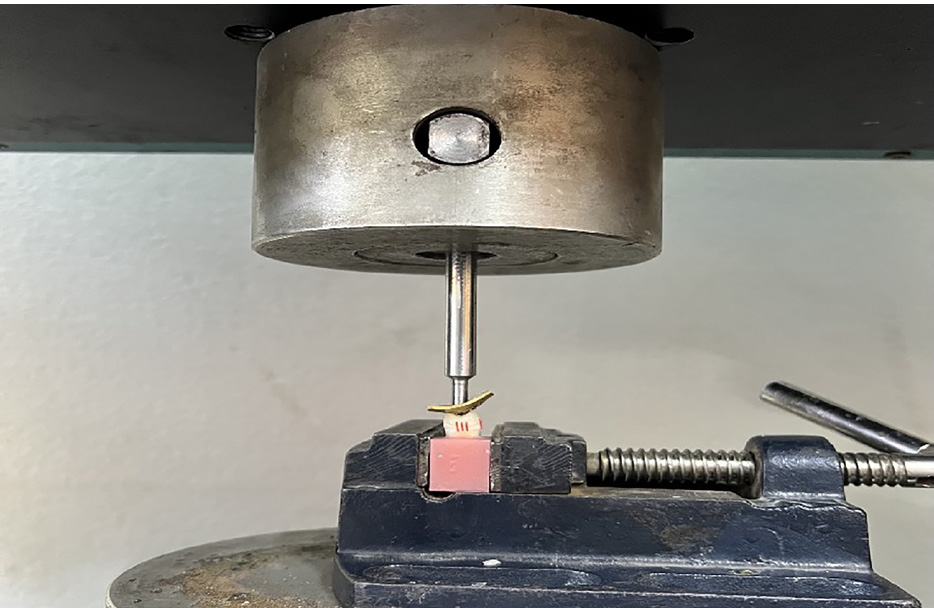
Fracture strength test.

The fracture strength of the overlay restorations was recorded through the use of a single load-till-failure test conducted in an electronically-controlled universal testing unit (Laryee, China). A vertical load was applied to the center of each restoration by means of a round-end stainless steel indenter at a 0.5 mm/min crosshead speed. And to prevent direct contact-induced deformation, a 1-mm-thick rubber layer was positioned at the interface of the restoration and the indenter [16], as shown in Fig. 3. All the samples underwent loading till failure occurred, and the measurements were documented in Newtons (N).

- Failure mode analysis

The failure mode of the samples was independently evaluated by both authors, each blinded to the group allocation to minimize potential observational bias. Discrepancies between evaluations were resolved through discussion to reach consensus. A digital microscope (Dino-Lite capture 2.0, version 1.3.6., Taiwan) under ×70 magnification was used to assess failure modes according to Burke’s classification ( Table 1).

- Statistical analysis

SPSS (version 22) was used for data analysis. The Shapiro–Wilk test at the 0.05 significance level was conducted to assess the normality of the data distribution, and one-way ANOVA was conducted to evaluate the statistical significance of the mean fracture load difference across the five groups.

## Results

**Figure 4:**
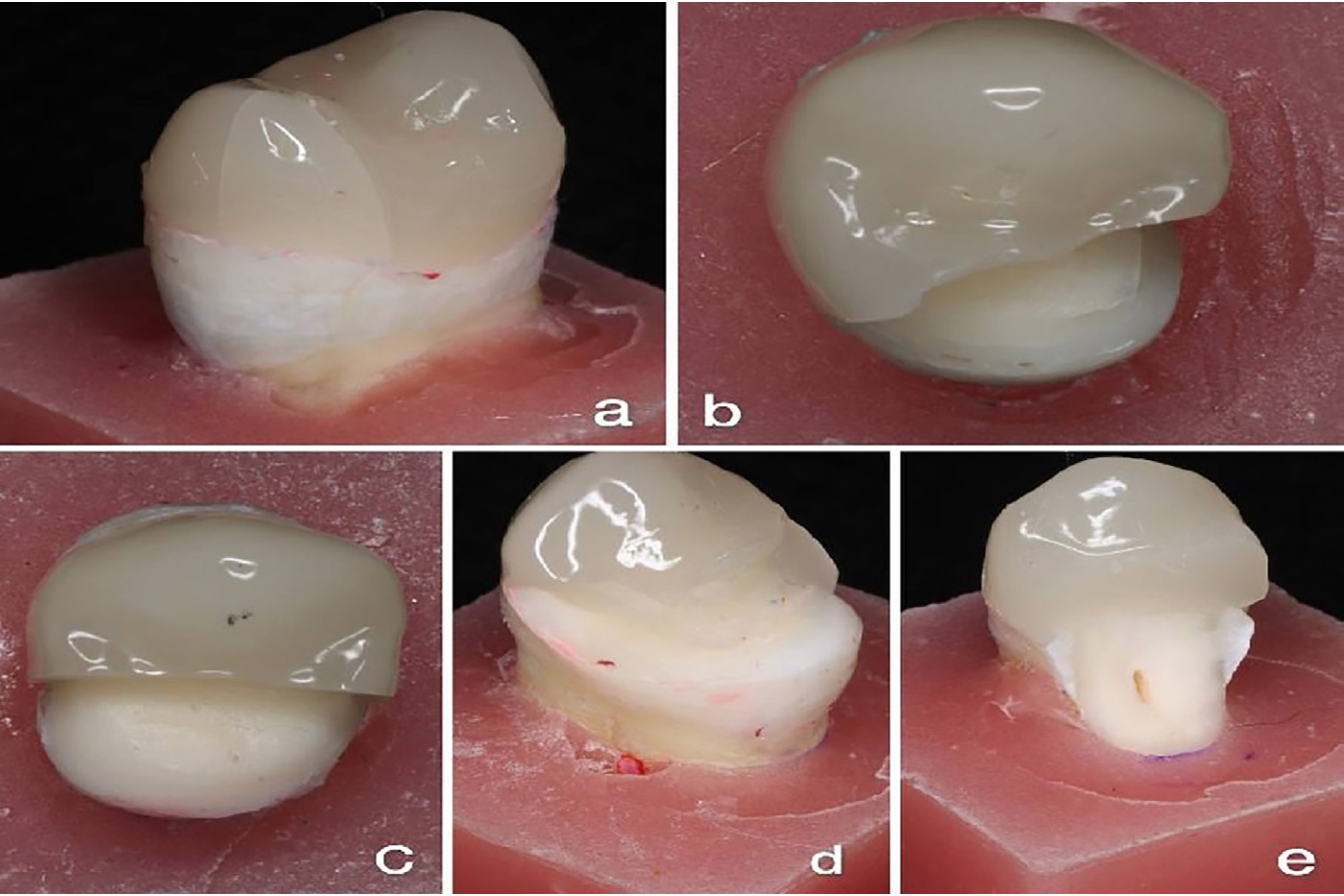
Failure modes of the different groups:(a) Code I failure mode;(b) Code II failure mode;(c) Code III failure mode;(d) Code IV failure mode;(e) Code V failure mode.

The mean fracture strength values were as follows: DDS group (1575.5 ± 187.7 N), IDS group (1545.6 ± 280.2 N), IDS + RC (1590.1 ± 247.4 N), IDS + SFRC (1549.8 ± 222.5 N), and IDS + Ribbond fiber group (1550.1 ± 272.9 N). The highest value was observed in the IDS + RC group, while the lowest was in the DDS group. However, one-way ANOVA test revealed no statistically significant differences among the experimental groups (*p* = 0.99). Descriptive statistics, including mean, standard deviation, minimum, and maximum, for all the groups, are shown in  Table 2. The Shapiro–Wilk test confirms the normality of data (*p* > 0.05). The modes of failure are outlined in  Table 3 and Fig. 4.

## Discussion

This research assessed the failure mode and fracture resistance of lithium disilicate overlay restorations reinforced with different biobase techniques. This investigation focused on comparing the performance of overlays with various fiber-reinforced biobases to that of overlays with conventional bonding techniques. To our knowledge, this study provides the first direct comparison of these five biobased approaches in the context of indirect lithium disilicate overlay restoration.

The results disclosed no significant difference in the fracture strength across the experimental groups (*p*>0.05). However, restorations with fiber-reinforced biobases (Groups D and E) resulted in more favorable failure modes than those in the non-reinforced ones (A, B, and C).

The mean fracture strength values across all the groups, ranging from 1545 to 1590 N, exceed the typical masticatory force generated during functional occlusion in the posterior region (ranging from 300 to 600 Newtons)[14], suggesting that lithium disilicate restorations, irrespective of the biobase technique used, possess sufficient strength to withstand functional masticatory loads. Our findings conform with earlier studies confirming the high fracture resistance of lithium disilicate, making it suitable for indirect restorations [17]. Besides the inherent strength of lithium disilicate, the high fracture strength values noticed in this investigation may be ascribed to several influencing factors, involving the preparation design, the cementation material choice, and the thickness of the restoration [18]. Particularly, a full-bevel preparation design was used, which is documented for its ability to increase the fracture strength [19]. Also, preheated composite for usage as cementation material was identified for its ability to enhance fracture resistance and bond strength [20]. Moreover, the restoration’s thickness surpasses the minimal mandated thickness for optimal load-bearing capacity under masticatory loads.

Despite the lack of significant differences in the fracture strength, the failure modes were remarkably affected by the fiber reinforcement. Specifically, restorations with fiber-reinforced biobases (Groups D and E) displayed more favorable failure modes than the non-reinforced groups (A, B, and C), which revealed more catastrophic failure modes. This proposes that fiber reinforcement, although not affecting fracture strength, can enhance total clinical performance by modifying fracture behavior and decreasing the severity of the failure.

This research’s results concur with those of Garoushi *et al*. [1,21], who confirmed that the inclusion of SFRCs, such as EverX flow beneath lithium disilicate overlay restorations, did not impact the load-bearing capacity; still, it efficiently shifted the failure mode to a more favorable pattern by facilitating energy absorption and hindering crack propagation.

The reinforcement effect observed in this research can be ascribed to the structural properties of the used fibers. Specifically, polyethylene Ribbond fibers, which are used in Group E, are acknowledged for their low elastic modulus, enabling them to absorb and distribute stress more effectively throughout the restoration. This stress-dissipating property is critical in mitigating the brittleness of lithium disilicate materials and decreasing the risk of catastrophic failure [22]. Embedding these fibers within a flowable composite base may also lower the modulus of elasticity of the tooth, possibly alleviating the residual stress [23]. These fibers demonstrate mechanical behavior akin to that of the dentin‒enamel complex, thus granting structural support to the overlay restoration and contributing to the inhibition of crack progression. This, in turn, reduces the chance of irreparable fractures. The noted reinforcement impact can be credited to the structural characteristics of the LWUHMWPE Ribbond fibers, which encompass a locked stitch interwoven framework with nodal intersections. This unique architecture facilitates intrinsic stress dissipation and energy absorption [12-14]. Fildisi *et al*. [24]reported that embedding Ribbond fibers beneath indirect overlay restorations enables a safer failure mechanism for fiber-reinforced restorations than their non-reinforced counterparts do.

In Group D, where short-fiber-reinforced composites (SFRCs), such as Ever X Flow, were utilized, the material revealed a high fracture toughness (2.8 MPa m1/2), similar to that of dentin. This might enable the SFRC to absorb stress comparable to DEJ and reduce stress transmission to the underlying tooth structures, hence preventing crack propagation and contributing to more favorable failure modes [14,25].

These outcomes agree with those of Magne *et al*. [26], who demonstrated that the utilization of a SFRC base (Ever X flow) improves the failure mode of indirect inlay restorations. Likewise, Otero *et al*. [27] reported that integrating an SFRC base (EverX flow) positively influenced the failure modes of cracked teeth restored with endocrown restorations. Contrariwise, Rocca *et al*. [28]stated that incorporating bidirectional or short random fiber-reinforced composites does not enhance failure modes.

The observed mechanical behavior of these fibers complies with the biomimetic dentistry principles, which underlines the usage of materials and approaches that simulate the natural biomechanics of enamel and dentine, thereby increasing the longevity and durability of dental restorations.

In contrast, the restorations in the non-reinforced groups (A, B, and C) exhibited more catastrophic failure patterns, highlighting the limitation of the inherent brittleness of lithium disilicate, which, despite its superior mechanical properties, is prone to fracture under excessive or repeated stress applications. The mismatch in modulus of elasticity of lithium disilicate (95 GPa) and dentine (11–19 GPa), coupled with the relatively low fracture toughness of the material (2.0–2.5 MPa m½), may contribute to stress accumulation within the restoration. This discrepancy in mechanical properties limits the ability of the restoration to deform plastically to the same extent as the underlying tooth structure, leading to crack initiation within stress-concentrated and load-bearing areas. Under dynamic loading conditions, these cracks may propagate, coalesce, and ultimately result in structural deterioration and catastrophic failure [29,30].

This study has several limitations. First, the Absence of periodontal ligament stimulation, which may restrict the applicability of the outcomes to real clinical scenarios, and the relatively large thickness of overlay restoration could have potentially masked the effect of the underlying reinforcements, making it difficult to completely check their effect. Moreover, the use of static loading in the fracture strength test may not fully replicate the dynamic masticatory forces typically encountered *in vivo*. The use of cyclic loading in future studies could offer a more clinically relevant evaluation of the material’s performance. Furthermore, a 1-mm biobase thickness may not have been optimal for entirely expressing the reinforcement potentials of the fibers, and future research could explore various biobase thicknesses to determine their optimum use.

## Conclusions

• All biobase techniques used in this study resulted in overlay restorations with sufficient strength to endure normal masticatory forces.

• The addition of fiber-reinforced biobases improved the failure mode.

These findings propose that fiber reinforcement could potentially improve the longevity and reparability of indirect lithium disilicate overlay restorations, rendering them a promising choice for restoring structurally compromised teeth. However, clinical investigations are required to verify the long-term effectiveness and real-world performance of these findings.

## Figures and Tables

**Table 1:** Burke classification of the failure mode.

Fracture mode	Description
Code I	Minimal fracture or crack in the crown
Code II	Less than half of the crown lost
Code III	Crown fracture through the midline (half of the crown displaced or lost)
Code IV	More than half of the crown lost
Code V	Severe fracture in the crown and/or tooth

**Table 2:** One-way ANOVA and descriptive data for the fracture strength.

Descriptive statistic					One-way ANOVA					
Group	Mean	SD	Min.	Max.		Sum of squares	DF	Mean square	F	Sig.
A (DDS)	1575.5	187.787	1305	1791	Between groups	15310.280	4	3827.570	.064	.99
B (IDS)	1545.6	280.268	1100	1915	Within groups	2691364.3	45	59808.096		
C (IDS + RC)	1590.1	247.421	1208	1840	Total	2706674.580	49			
D (Ever X flow)	1549.8	222.514	1208	1925						
E (Ribbond)	1550.1	272.941	1190	1980						

DDS: Delayed dentin sealing; IDS: immediate dentin sealing; Max.: maximum; Min.: minimum RC: resin coating; SD: standard deviation; Sig: significance.

**Table 3:** Failure modes of all the groups.

Group	Code I (%)	Code II (%)	Code III (%)	Code IV (%)	Code V (%)	Total
A (DDS)	-	1 (10%)	2 (20%)	2 (20%)	5 (50%)	10 (100%)
B (IDS)	-	2 (20%)	1 (10%)	3 (30%)	4 (40%)	10 (100%)
C (IDS + RC)	-	2 (20%)	2 (20%)	2 (20%)	4 (40%)	10 (100%)
D (Ever X flow)	-	4 (40%)	4 (40%)	-	2 (20%)	10 (100%)
E (Ribbond)	1(10%)	5 (50%)	2 (20%)	-	2 (20%)	10 (100%)

DDS: Delayed dentin sealing; IDS: immediate dentin sealing; RC: resin coating

## Data Availability

The datasets used and/or analyzed during the current study are available from the corresponding author.
